# Erratum: Abma, T., et al. Sowing Seeds to Harvest Healthier Adults: The Working Principles and Impact of Participatory Health Research with Children in a Primary School Context. *Int. J. Environ. Res. Public Health* 2020, *17*, 451

**DOI:** 10.3390/ijerph17093252

**Published:** 2020-05-07

**Authors:** Tineke Abma, Sarah Lips, Janine Schrijver

**Affiliations:** 1 Department of Medical Humanities, University Medical Centre, 1081 HV Amsterdam, The Netherlands; s.lips@amsterdamumc.nl; 2 Art Collective B.A.D, 3082 MG Rotterdam, The Netherlands; janineschrijver@xs4all.nl

The authors wish to make the following corrections to the above-mentioned published paper [1]:

1. During production, an error occurred in the layout of Figure 1 ‘Learning impact of KLIK, as self-assessed by the children’ on page 6. As a result, the clarifying text did not align well with the bar chart. The corrected figure should be as depicted below.
2. In the supplementary materials, an incomplete and partly Dutch version of Table S1 ‘Activities with the children in the KLIK-program 2016–2019’ (ijerph-668898-Table 1.pdf) was accidentally provided in the original version of our article [1]. This should be replaced by ‘Table S1_Activities KLIK-project 2016–2019_Corrected’.

The authors would like to apologize for any inconvenience caused to the readers by these changes.

## Figures and Tables

**Figure 1:**
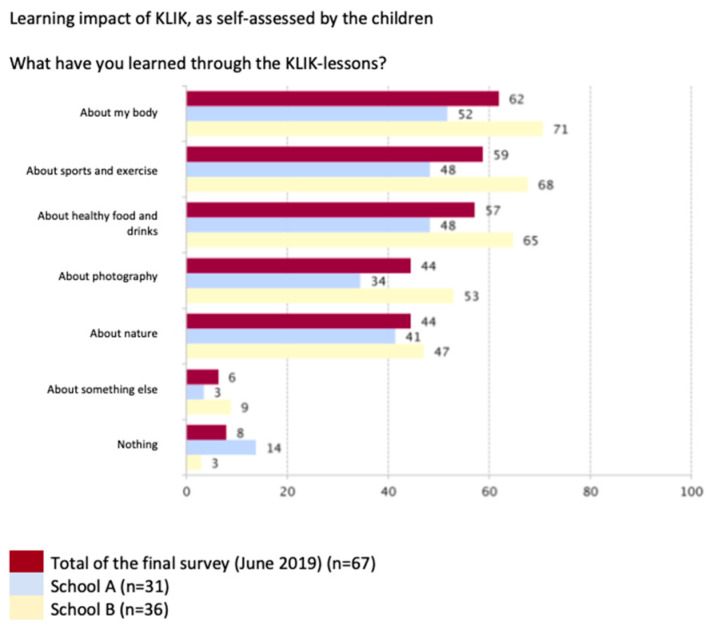
Learning impact of KLIK.
