# Supplementary material for: Sowing Seeds to Harvest Healthier Adults: The Working Principles and Impact of Participatory Health Research with Children in a Primary School Context
Source: Int J Environ Res Public Health. 2020 Jan 10;17(2):451. doi: 10.3390/ijerph17020451 (PMC7014369; doi:10.3390/ijerph17020451)
Supplement: Supplementary file 1 [file ijerph-17-00451-s001.zip › ijerph-668898-supplementary/ijerph-668898-table 1.pdf]

Table 1. Activities with the children in the KLIK-program 2016 - 2019.

School year 2016-2017		School year 2017-2018		School year 2018-2019	
**Ongoing throughout the years: observations, field notes, evaluative discussions with all participants**					
**Activity**	**Description**	Activity	Description	Activity	Description
Wild pick walk	Walk through the neighborhood, during which the children learned about and gathered eatable wild plants. Afterwards they learned to make fruit drinks ('smoothies'), mixing in the plants they had picked.	KLIK's Research/Investigatory Agency	A group of children selected from the participating classes gathered once a week to discover, formulate, investigate and answer their own research questions. In this process, various scientific and artistic methods were explored (like making a film, photography, interviewing, and more). Later, the results were presented at school.	Visiting museum Corpus	Visit of a museum in which the children made a journey through the human body, followed by a written and drawn evaluation of their experiences and impressions
Series of lessons on photography	Series of lessons in which photography was explained, as a means to observe your environment and express how you experience this, followed by a 'Photography walk', during which the children photographed what they thought noticeable in their neighborhood environment. They then took home the camera and made pictures of their meals in their home environment. Finally, the photographs taken by the children were shown in class, and the children reflected on these pictures.	Series of XXX lessons on movement, exercise, like Capoeira and a 'Sportlab'	We hebben aantal lessen gedaan bij de vier deelnemende klassen van de OBS en Triangel over wat er in je lichaam gebeurt als je beweegt. Tijdens de eerste KLIK les hebben we een Capoeira docent ingehuurd om een les te geven, en de tweede en derde les dingen over het Sportlab: wat voel je als je beweegt. Bij de vervolg les gingen we inhoudelijk wat dieper in op de onderwerpen, en koppelden we een aantal foto's terug. Tips en trics om meer te bewegen tekeningen gemaakt hoe hun ideale speeltuin eruit ziet en aangegeven wat een goed en slechte speelplek is	Plants laboratory	Introduction on caring, attention and growth
Algenbak maken + wedstrijd: wie heeft de groenste bak + 2 weken later algenbak oogsten & smoothie maken		Series of courses on mindfullnes		Body laboratory	Introduction on caring, attention and health How to train strength / endurance / flexibility
Proeflab Proeflab maken in b.a.d. en klassen meenemen daarheen en blind laten proeven, eten met gekke kleuren, koud&warm , gekookt, ongekookt, en textuur. Drietallen kinderen:		Jan 2017: Klikonderzoeksbureau met kinderen van O.B.S. Charlois	vragen formuleren en onderzoeken gaat voor deze kinderen te ver. De foto's die ze gemaakt hebben worden in de KLIK krant verwerkt<br><br>filmpjes die de kinderen van de Triangel bij het KLIKonderzoeksbureau gemaakt	Evaluative interviews	Group interviews with the children about their experiences with caring for their plants, bodies and for themselves in a broader sense

om en om proeven, foto maken tijdens proeven en ander schrijft op.			hebben zijn tijdens het B.a.d café en in de klassen vertoond		
OBS 1+2: Workshop journalistiek over kennis overbrengen andere kinderen		ideeën bus		Evaluative writings, drawing, …	
OBS 1+2: Les door ons over voedingsprincipes en journalistieke vaardigheden: maak een poster met elkaar om in de school te hangen, met de basisprincipes. Na les kinderen uitnodigen voor Kinderpersbureau	Begin juni is de eerste KLIK krant, gemaakt door de kinderen zelf, uitgereikt aan de kinderen van de vier klassen (zie beeldmateriaal). De KLIK krant geeft een verslag van waar de kinderen aan hebben gewerkt in 2016-2017, en wat ze hebben geleerd. Op de bijeenkomst lazen een paar kinderen voor het publiek een stuk voor dat zij hadden geschreven over wat zij hadden geleerd (oa hoe de spijsvetering werkt; smaken proeven; schijf van vijf; hoe je kinderen groenten kunt leren eten), en ook beantwoorden een paar kinderen vragen over wat zij geleerd hebben van KLIK. Ook twee docenten kwamen naar voren op het podium om een paar vragen te beantwoorden,	Mindfulness training die door de trainers aandachttraining wordt genoemd		Making the KLIK newspaper	Children engaded in: doing (mutual) interviews; making photo's, drawings, comics; writing reports and personal impressions
OBS 1+2: Les voeding deconstrueren: wat zit er in…		diëtiste's van De Groene Appel hebben school breed in de groepen 3 tot en met 8 voedingsles gegeven		Preparing the final presentation	
Mei en juni 2017:<br>2x Les nog in te vullen voor de OBS -> werd ingevuld met KLIK-krant maken?		De O.B.S Charlois is zelf nog een aanvullend programma gestart, Smaaklessen.	mooi aansluit bij KLIK. Deze lessen worden door de docenten zelf gegeven	Presenting the KLIK project and newspaper	Including:<br>- collaboratively constructing a tower
KLIKkrant schooljaar 2016-2017	Preparing for presenting the newspaper, telling about what they learned and how they experienced this	KLIKkrant schooljaar 2017-2018		Preparing the final presentation	

presentatie van de KLIK krant	presentatie van de KLIK krant	Presenting the KLIK project and newspaper
	(Najaar 2017 geen activiteiten?)	
