# Supplementary figures and images for: Sowing Seeds to Harvest Healthier Adults: The Working Principles and Impact of Participatory Health Research with Children in a Primary School Context
Source: Int J Environ Res Public Health. 2020 Jan 10;17(2):451. doi: 10.3390/ijerph17020451 (PMC7014369; doi:10.3390/ijerph17020451)

Graph 1\_Children's appreciation of the KLIK-lessons

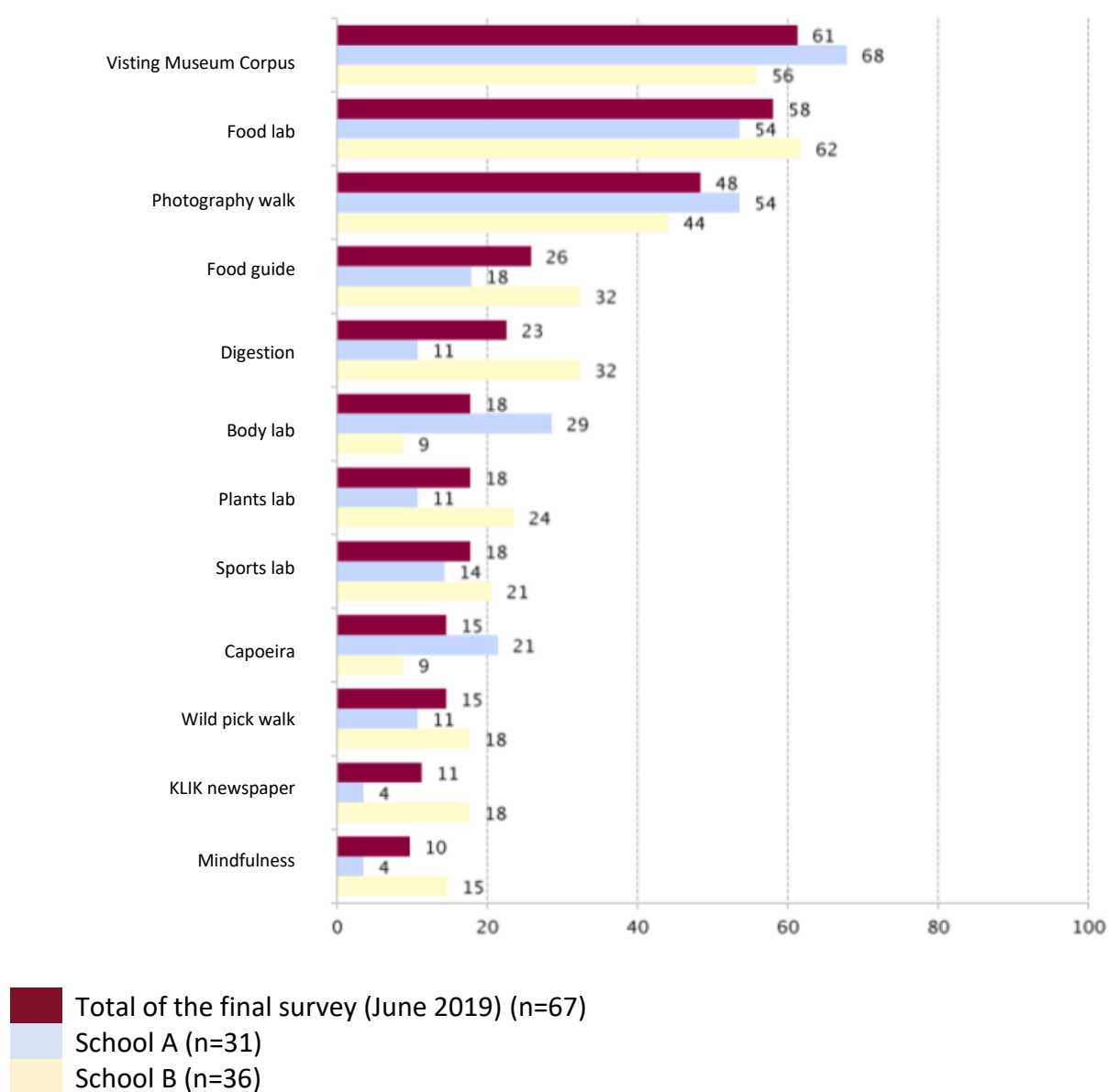

Supplement: Supplementary file 1 [file ijerph-17-00451-s001.zip › ijerph-668898-supplementary/ijerph-668898-graph 1.pdf]
